# Maize seeds forecasting with hybrid directional and bi‐directional long short‐term memory models

**DOI:** 10.1002/fsn3.3783

**Published:** 2023-11-09

**Authors:** Hakan Isik, Sakir Tasdemir, Yavuz Selim Taspinar, Ramazan Kursun, Ilkay Cinar, Ali Yasar, Elham Tahsin Yasin, Murat Koklu

**Affiliations:** ^1^ Department of Electric‐Electronic Engineering Selcuk University Konya Turkey; ^2^ Department of Computer Engineering Selcuk University Konya Turkey; ^3^ Doganhisar Vocational School Selcuk University Konya Turkey; ^4^ Guneysinir Vocational School Selcuk University Konya Turkey; ^5^ Department of Mechatronic Engineering Selcuk University Konya Turkey

**Keywords:** classification, forecasting, hybrid CNN, maize seeds, purification

## Abstract

The purity of the seeds is one of the important factors that increase the yield. For this reason, the classification of maize cultivars constitutes a significant problem. Within the scope of this study, six different classification models were designed to solve this problem. A special dataset was created to be used in the models designed for the study. The dataset contains a total of 14,469 images in four classes. Images belong to four different maize types, BT6470, CALIPOS, ES_ARMANDI, and HIVA, taken from the BIOTEK company. AlexNet and ResNet50 architectures, with the transfer learning method, were used in the models created for the image classification. In order to improve the classification success, LSTM (Directional Long Short‐Term Memory) and BiLSTM (Bi‐directional Long Short‐Term Memory) algorithms and AlexNet and ResNet50 architectures were hybridized. As a result of the classifications, the highest classification success was obtained from the ResNet50+BiLSTM model with 98.10%.

## Graphical abstract

A special dataset was created to be used in the models designed for the study. The dataset contains a total of 14,469 images in four classes. Images belong to four different maize types, BT6470, CALIPSO, ES_ARMANDI, and HIVA, taken from the BIOTEK company. AlexNet and ResNet50 architectures, with the transfer learning method, were used in the models created for the image classification. In order to improve the classification success, LSTM (directional long short‐term memory) and BiLSTM (Bi‐directional long short‐term memory) algorithms and AlexNet and ResNet50 architectures were hybridized.

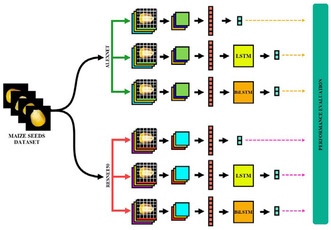

## 1 INTRODUCTION

The purity of products is an important factor in the marketing of grain products. For this reason, farmers need to know the type of seed they use in order to grow and harvest the right product (Chen et al., 2010). Maize (*Zea mays*) is a cereal product that is widely grown in numerous regions of the world today and has a wide usage area (Ambrose et al., 2016). In addition to being used as human food and animal feed, it is also used in many industrial products (Avuçlu et al., 2023). After harvesting, maize seeds are likely to be confused with different maize cultivars in processes such as logistics and storage. This situation leads to economic losses along with the deterioration of the cultivars' purity. For these reasons, classification has significance in determining the pre‐sowing quality of maize (Zhao et al., 2018).

Image processing techniques and analysis methods can be applied to understand the features of images or to make quantitative measurements of objects on images (Loddo et al., 2021). Image processing techniques, which can be successfully applied in many classification problems in this way, are popularly used in various fields of agriculture such as legumes, fruit, plant leaves, and plant diseases (Ashqar et al., 2019; Cinar & Koklu, 2019; Koklu et al., 2021; Yasar et al., 2015). In the studies, different methods and techniques have been developed for classification problems with both deep learning methods and machine learning methods.

The agricultural sector constantly needs new technologies and methods to produce food in a sustainable and efficient manner. Maintaining the purity of seeds is a basic requirement to increase production quality and yield. This study aims to help farmers make more informed decisions in seed selection and production processes by using advanced artificial intelligence and image processing techniques that enable accurate classification of maize seeds. In addition, by presenting new models and methods to the literature, it expands the existing knowledge on the classification of maize seeds and offers more effective solutions. This study contributes to current issues such as the application of technology‐based innovations in agriculture and the improvement of food production.

In this study, images belonging to four types of maize seeds were classified; BT6470, CALIPOS, ES_ARMANDI, and HIVA. The contribution of this study to the literature is as follows:

- A dataset consisting of 14,469 images of four maize seed cultivars was generated.
- Hybrid models were created by using AlexNet and ResNet50 architectures with LSTM and BiLSTM.
- Six different models have been proposed to classify the images of maize seeds.
- New models have been introduced to the literature thanks to the contribution of the proposed hybrid models to classification success.

The remainder of the study is designed as follows. In the second chapter, the literature on the subject is reviewed. In the third chapter, the methods used in the study, the dataset, and the methods used for performance measurements are explained. In the fourth chapter, experimental studies are explained. In the fifth section, the results are discussed. In the last chapter, giving the results of the study, suggestions are presented.

## 2 RELATED WORKS

Numerous studies have been carried out on maize, which has a very large cultivation area around the world. In the studies on maize, it is seen that artificial intelligence, machine learning, and deep learning methods are frequently used in different problems such as disease diagnosis or quality and cultivar classification. Studies on the classification of maize cultivars in the literature are summarized below.

A new technique has been developed from visible and near‐infrared hyperspectral images in a study aiming to classify maize cultivars based on morphological and texture features. By using support vector machines (SVM) and partial least squares‐discriminant analysis (PLS‐DA) models for the classification of cultivars, a classification success of 96.3% and above was obtained from the SVM model (Yang et al., 2015).

In another study in which images of maize seeds are used, it was suggested to perform the classification by extracting morphological features such as color, texture, and shape from the obtained images. For this classification problem, genetic algorithms and Support Vector Machine was utilized within the scope of the study. The classification success obtained as a result of the study is 94.4% (Zhao et al., 2011).

Within the scope of the study in which a fast and non‐destructive classification of four different maize seed cultivars was performed through hyperspectral imaging, images obtained at a wavelength of 450–979 nm were classified by DCNN (deep convolutional neural network), k‐NN (K‐nearest neighbors), and SVM classification models. As a result of the study, it was stated that DCNN gave better results compared to other models with a performance success of 94.4% (Zhang et al., 2021).

In the study, in which the maize seeds covered with the help of a hyperspectral camera were classified based on cultivars, logistic regression (LR) and SVM from machine learning architectures and convolutional neural network (CNN), recurrent neural network (RNN), and LSTM from deep learning architects were used for the creation of a classification model. Besides, the classification of extracted features with the help of principal component analysis (PCA) was compared. It was stated that the classification of maize seeds obtained from hyperspectral images was more successful than the images with extra features (Zhang et al., 2020).

In the study where the deep learning method GoogLeNet and machine learning method SURF+SVM were used for quality classification of seeds, 95% and 79.2% accuracy performances were obtained, respectively. The results of the study demonstrated that increasing net depth also increases the accuracy (Huang et al., 2019).

A new network was used for the classification of maize kernels with the help of Mask R‐CNN, one of the well‐known deep learning methods for image segmentation. The average accuracy rate of the deep learning model proposed within the scope of the study, in which maize were classified as good, dirty, and bad, was reported as 95.6%. In this study, Mask R‐CNN, VGG16, and ResNet50 models were compared (Velesaca et al., 2020).

In another study, using the machine developed to distinguish the maize cultivars, maize images were obtained through a double camera. The obtained images were classified via SVM, artificial neural network (ANN), PCA, and ResNet architectures. The methods used were compared and it was seen that ResNet had the highest accuracy rate with 98.2% (Ni et al., 2019).

In the study, which presents a new CNN‐based approach to the classification of maize cultivars, ANN was classified by cubic SVM, quadratic SVM, weighted kNN, reinforced tree, bagged tree, and linear discriminant analysis (LDA) methods. It was reported that the features obtained by deep learning methods show better classification success than the simple features extracted. The classification results revealed that the CNN–ANN classifier, which reached 98.1% accuracy performance, is more successful than the other tested methods (Javanmardi et al., 2021).

When the literature is examined, there are many studies conducted with both machine learning and deep learning methods. A brief summary of the above‐mentioned studies on maize grain varieties is given in Table 1.

**TABLE 1:** Similar studies found in the literature.

Image	Method	Proposed model	Best accuracy	References
Visible and near‐infrared hyperspectral images	Morphological and texture	SVM PLS‐DA	96.3% (SVM)	Ashqar et al. (2019)
RGB images	Color, texture, and shape	SVM	94.4% (SVM)	Yang et al. (2015)
Hyperspectral imaging (wavelength of 450–979 nm)	Morphological and texture	DCNN k‐NN SVM	94.4% (DCNN)	Zhao et al. (2011)
Hyperspectral images	PCA Deep features	LR SVM CNN RNN	Accuracy over 90% (all models)	Zhang et al. (2021)
RGB images	Deep features	GoogLeNet SURF+SVM	95% (GoogLeNet)	Zhang et al. (2020)
RGB images	Image segmentation		Average 95.6%	Huang et al. (2019)
RGB images	Deep features	SVM ANN PCA ResNet	98.2% (ResNet)	Velesaca et al. (2020)
RGB images	Deep features Simple features	ANN + Cubic SVM Quadratic SVM weighted k‐NN reinforced tree Bagged tree LDA	98.1%	Ni et al. (2019)

## 3 MATERIALS AND METHODS

**FIGURE 1:**
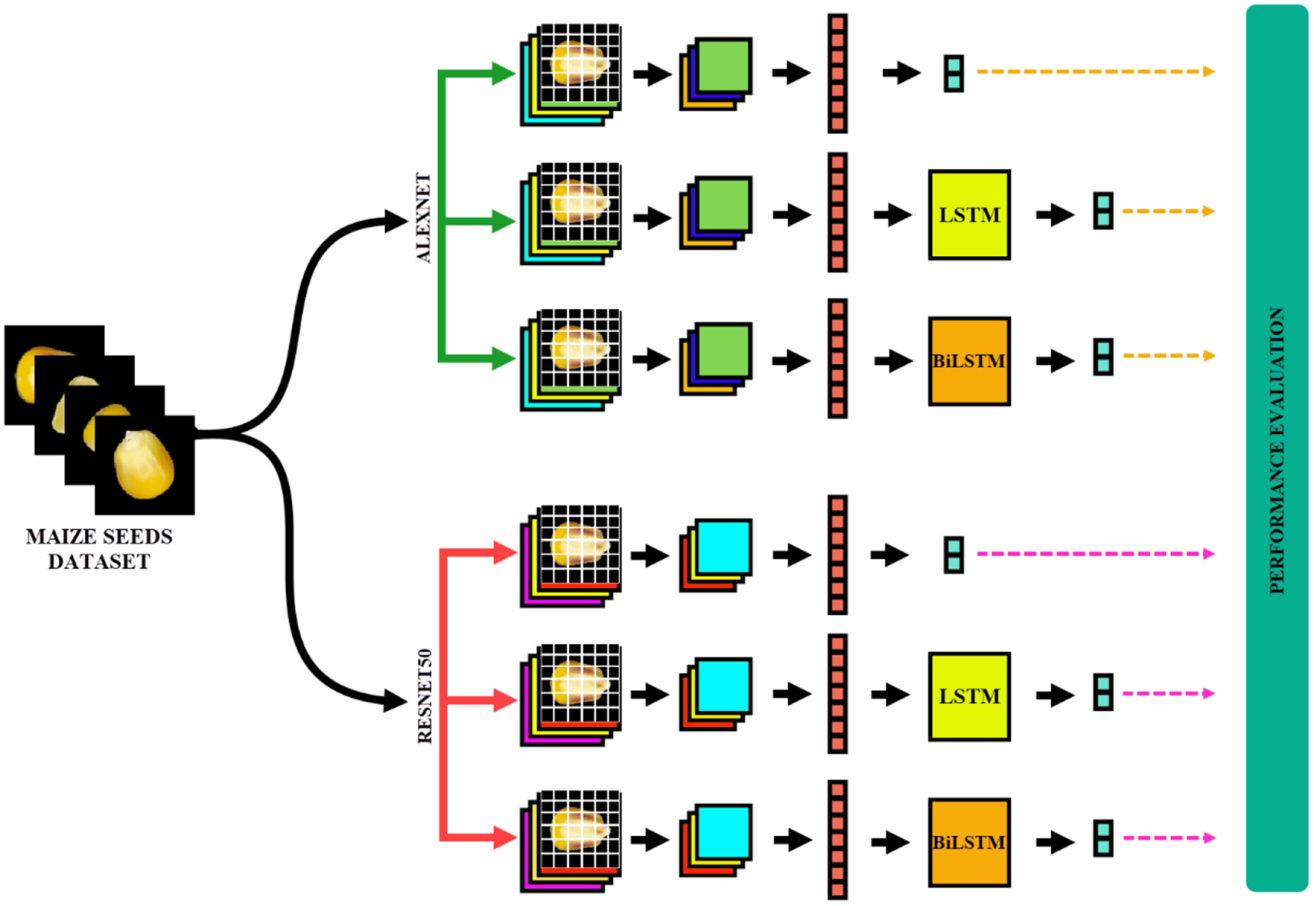
Flow chart of maize seeds classification.

In this study, six different CNN‐based models have been proposed. AlexNet+LSTM and AlexNet+BiLSTM hybrid models were created by hybridizing AlexNet and this model. The ResNet50 CNN model was utilized to be able to make a comparison with another model with a different number of layers. As with AlexNet, the ResNet50 model is hybridized with LSTM and BiLSTM. Figure 1 gives the flow chart showing the use of obtained models in classification.

In this chapter, detailed information about the dataset and acquisition of the dataset is given. CNN and transfer learning, and AlexNet, ResNet50, LSTM, and BiLSTM used in the study will be briefly explained. In addition, information about the confusion matrix and performance metrics used in the models' performance analysis will be presented.

### 3.1 Image acquisition and dataset

**FIGURE 2:**
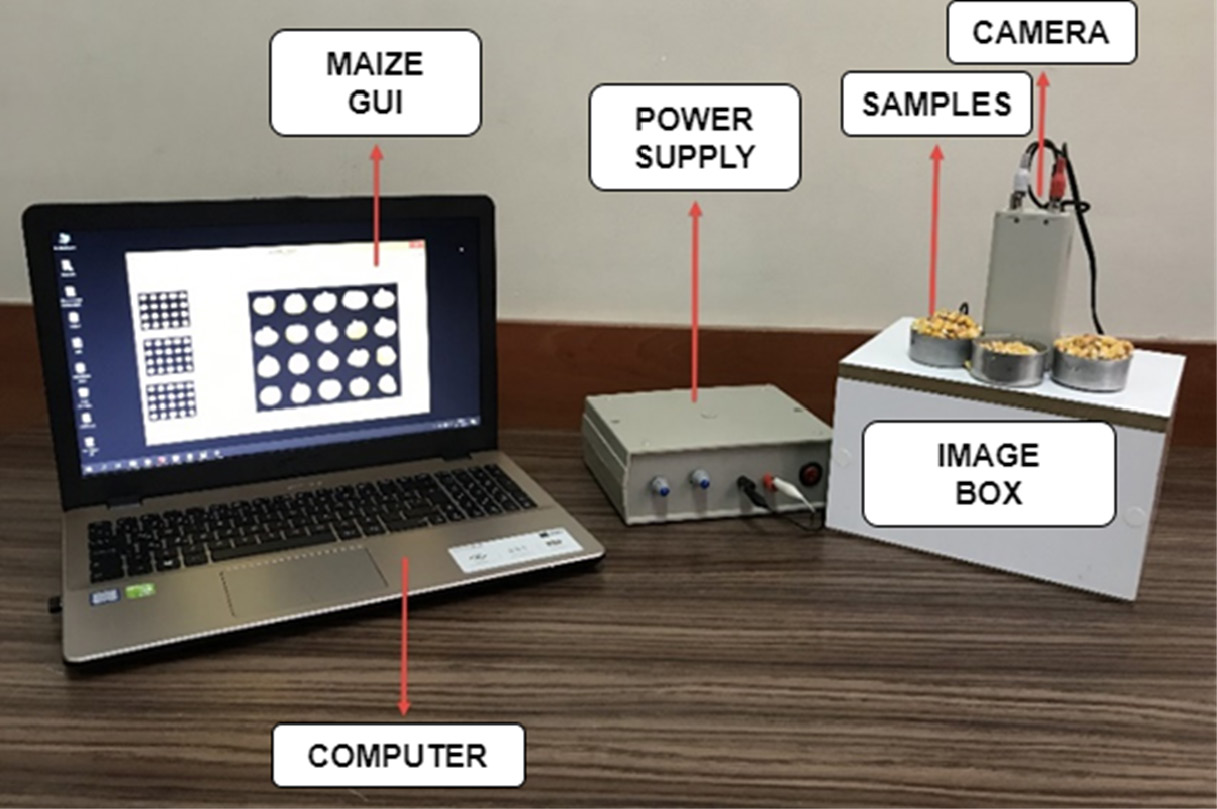
Mechanism established for obtaining images of maize kernels.

Within the scope of this study, a dataset containing a total of 14,469 images was created from four different maize cultivars that are widely produced in Turkey. In order to generate the dataset, a mechanism consisting of a closed box equipped with an LED light system and a camera placed on this box was created. Figure 2 shows the created image acquisition mechanism (Kishore et al., 2022).

**FIGURE 3:**
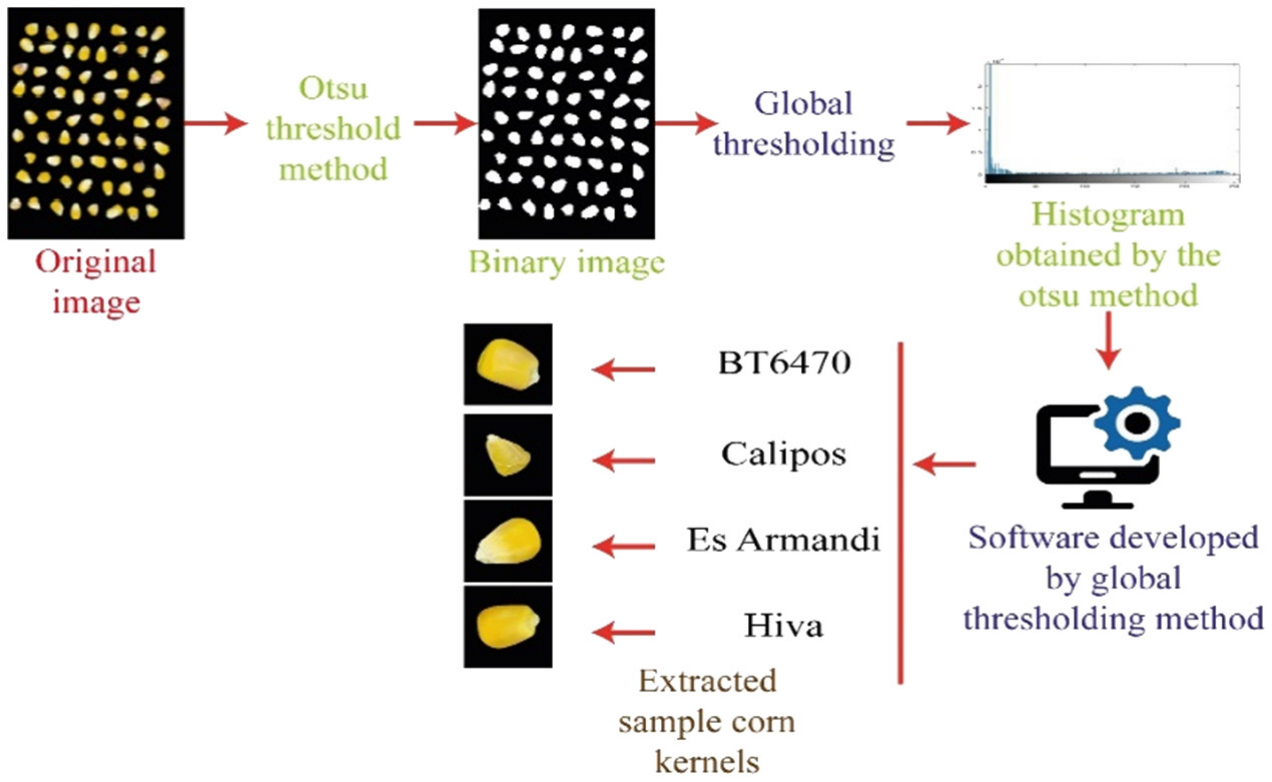
Acquisition of individual maize cultivars from original images by pre‐processing.

**FIGURE 4:**
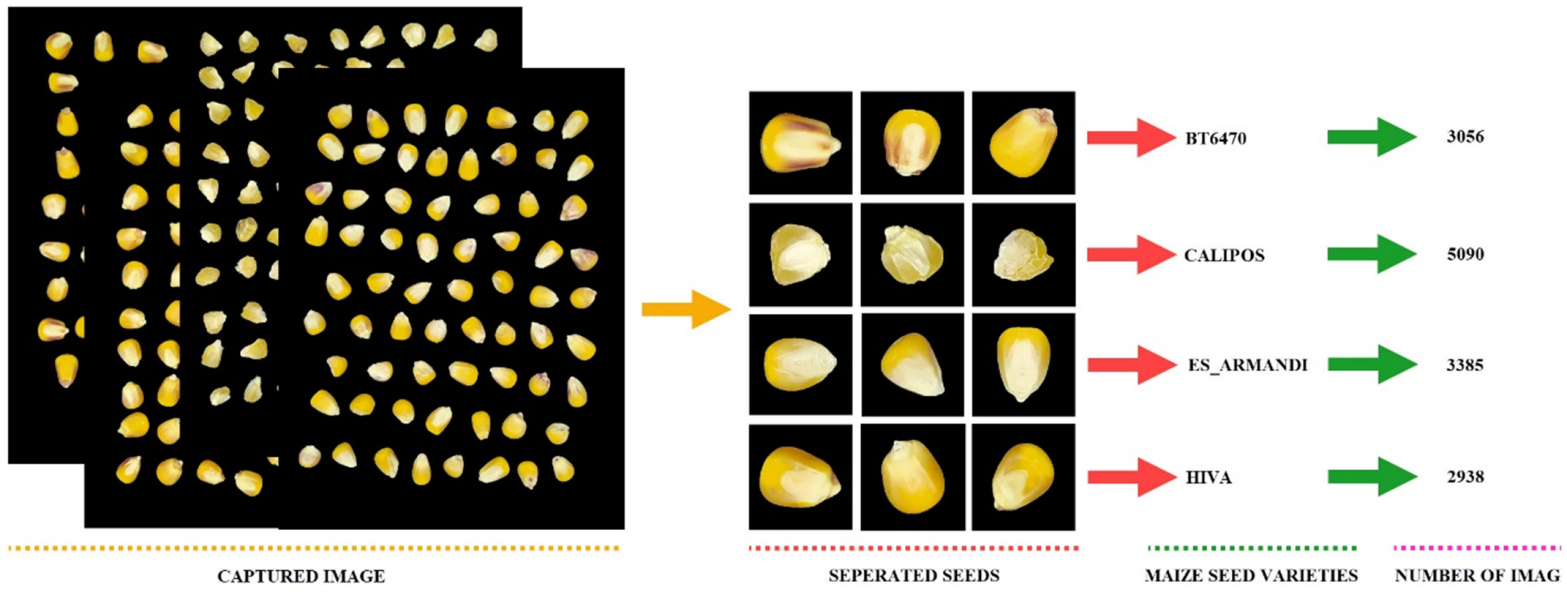
Number of maize images according to cultivars.

Pure maize kernel images were obtained by preventing shadow formation in the box illuminated with LED lights. It is aimed to reduce noise in image processing by setting the backgrounds of images taken from a distance of 15 cm to black. In order to obtain the images, maize kernels of the same cultivars were placed in the box together. In the next step, maize kernels were segmented to obtain each grain image. The image of each maize kernel was obtained in 350×350 sizes. About 1 kg of sample from each maize cultivar was used. Since the kernels are in different sizes and weights for each cultivar, different numbers of images were obtained for each one (Kishore et al., 2022). While creating the dataset, random maize kernels were obtained by arranging them as symmetrically as possible on a black background. Choosing a shadowless and black background of the background color enables the image to be simply converted into a binary image with the Otsu method (Pramanik et al., 2015). Background and maize kernels have been transformed into binary images as distinctive from each other. Thanks to the software developed using the simple equalization (Wang et al., 2019) technique from the obtained binary image, the borders of the maize kernels were determined according to the white threshold value and extracted from the original image as 350×350 pixels, since the object grains are white and the background is black. The pre‐processing process of the image is given in Figure 3 in detail (Kishore et al., 2022). Figure 4 shows maize kernels number of maize kernels images of four different maize cultivars (Kishore et al., 2022).

### 3.2 Convolutional neural network (CNN)

CNN is a type of deep learning algorithm (Taspinar & Selek, 2020). With this algorithm, in addition to end‐to‐end image classification, it is also possible to obtain deep features from images (Bicakci et al., 2020). CNN is a kind of neural network consisting of convolutional, pooling, and activation layers. In the convolutional layer, kernels are applied to images. All outputs of the convolutional layers form the feature maps. In the activation layer, it is ensured that non‐linear data are included in certain intervals. In this way, the input data is normalized and the learning process in the hidden layers is accelerated (Yurttakal et al., 2021). In the architectures in this study, the process of reducing the size of the data from the feature map from the convolution layer is performed in the activation function rectified linear unit (ReLu) pooling layer. The features extracted by this process are reduced to significant features, ensuring that overfitting does not occur. After this stage, there are different pooling methods. Max, average, and sum pooling are some of these methods. Generally, average pooling is used as more effective features can be obtained. Average pooling was used in the architectures in this study. In order to get rid of the overfitting problem, generally, the dropout layer is used in CNN architectures (Bicakci et al., 2020; Taspinar et al., 2021a). This layer randomly discards some neurons in each iteration. In order to reduce the image features to one dimension, a flatten layer is used before the final layers (Ambrose et al., 2016; Aslan et al., 2020; Uyar et al., 2022). After this layer, the data enters the fully connected layer. The structure of this layer is the same as the neural network structure. In this layer, classification processes are performed as learning the image features by the network (Altay & Altay, 2023a; Unal et al., 2022).

### 3.3 Transfer learning (TL)

TL is utilized to create models with strong classification ability by fine‐tuning the pre‐trained model, with the small number of data contained in the dataset to be used (Butuner et al., 2023). The model, which is previously trained with a large amount of data and has a high capability of image classification, is retrained by using the target dataset (Yasar, 2023a). The similarity between the data on which the pre‐trained model is trained and the data contained in the target dataset is one of the main factors affecting the classification ability of the model (Koklu, Unlersen, et al., 2022). The pre‐trained model can successfully extract common and hidden features with the source dataset on the images in the target dataset. These advantages have made the use of CNN pre‐trained models quite popular in recent years (Koklu, Unlersen, et al., 2022). AlexNet and ResNet50, which are frequently used pre‐trained models in the literature, were used for feature extraction from images in the study.

#### 3.3.1 AlexNet

Proposed by Krizhevsky et al., the AlexNet model generally consists of eight layers. It has many advantages in image classification. The image size entering the network is 227×227×3. Color (RGB) images are supported by the network. The layers within the network are convolution where features are extracted, activation rectified linear unit (ReLu), maximum pooling, and normalization layers where dimensionality is reduced. The layers before the classification layer are the fully connected layer where the image features are learned. It can be used in different machine learning models by taking 4096 image features from the fully connected layer just before the Softmax layer (Unlersen et al., 2022).

#### 3.3.2 ResNet50

ResNet50, a deep learning architecture with more layers than AlexNet, contains 50 layers in total. Higher classification success can be achieved as the number of layers increases in datasets with a large amount of data. The image size entering the network is 224×224×3. RGB images are supported. As in other CNN architectures, it includes convolution in which features are extracted within the network, activation Rectified Linear Unit (ReLu), and maximum pooling and normalization layers where dimensionality is reduced. The layers before the classification layer are the fully connected layer where the image features are learned. It can be used in different machine learning models by taking 4096 image features from the fully connected layer just before the Softmax layer (Theckedath & Sedamkar, 2020).

### 3.4 Long short‐term memory (LSTM) and Bi‐directional long short‐term memory (BiLSTM)

**FIGURE 5:**
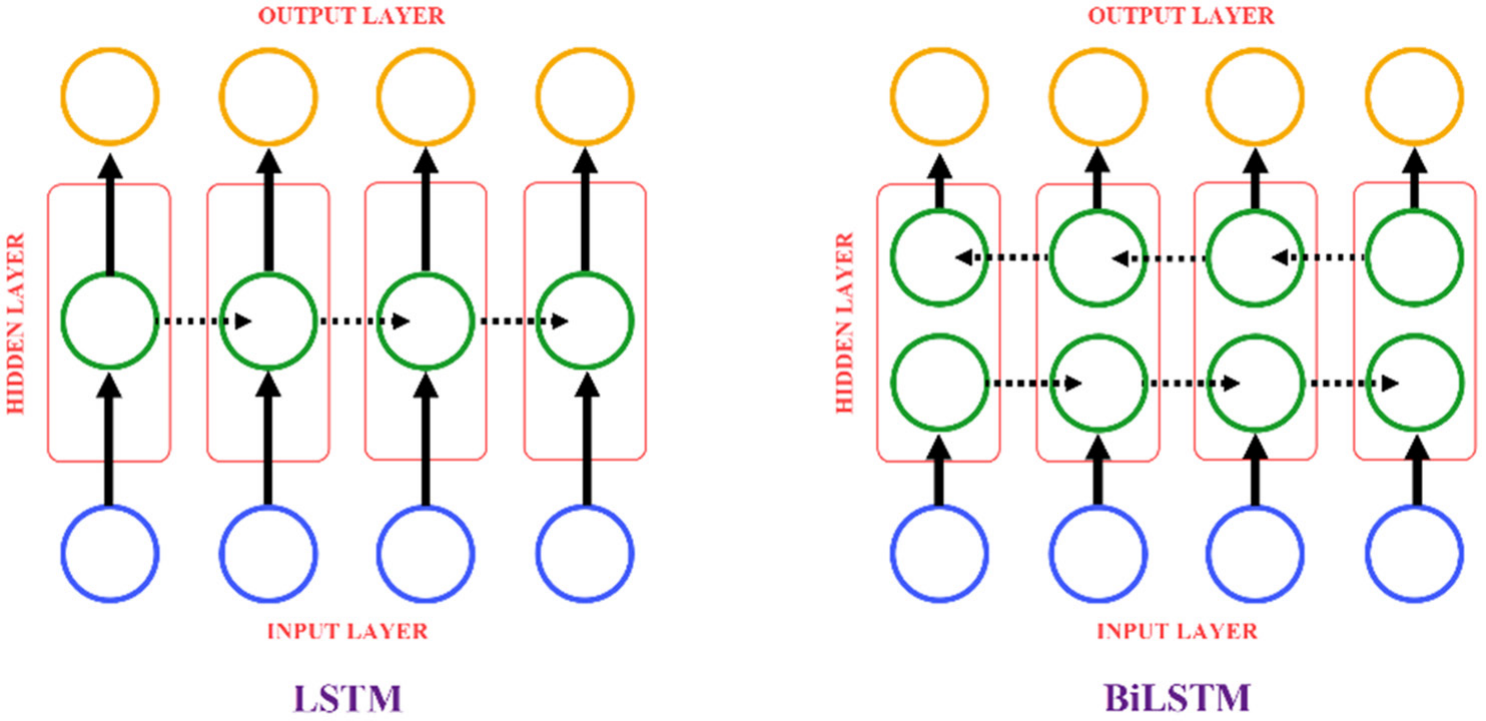
LSTM and BiLSTM architectures.

Recurrent neural networks (RNNs) are a type of neural network designed to perform the prediction of sequential data. Training and testing of RNN models differ from other machine learning methods since a different layout is introduced to the data. LSTM is a kind of RNN created to eliminate the instability (short‐term memory problem) in RNNs. LSTMs can learn and use the temporal dependency (Aslan et al., 2021). LSTMs have internal memories. In this way, it can perform the training and estimation by taking the input as a string, not with the current input to the network. LSTM networks differ from CNNs in that LSTM cells contain 3 gates (Golcuk et al., 2023). The LSTM cell contains the entrance gate, exit gate, and forget gate. Input gate updates the cell state. It compresses the data coming into the cell between −1 and 1 with the tanh activation function for the arrangement process. It is the gate where the information to be updated is decided. Forget gate is the gate where the decision is made about which information will be forgotten or kept. The information from the previous cell and the current information is given as input to the sigmoid activation function and the result is decided. Information that is 1 continues to be transmitted through the cell state, information that is 0 is forgotten. The output gate determines the input of the next cell. It is also used to make estimations. Previous information and current information are given as inputs to the sigmoid activation function. Then, the existing information on the cell state is given as an input to the tanh function (Vincent et al., 2020). As a result, the two results are multiplied and it is decided which information will be the input of the next cell. LSTM and BiLSTM architectures have the same way of operation. BiLSTM is a superimposed variant of LSTM. Transactions in LSTM are performed bidirectionally. In this way, BiLSTM can provide a high capacity for the learning (Koklu, Cinar, & Taspinar, 2022). Figure 5 gives the general representation of LSTM and BiLSTM architectures.

There are many cells within the LSTM and BiLSTM architectures. These cells enable classification by learning data features. They show high success, especially in time series data. The purpose of using it in this study is to increase the success performance by enabling more learning of the image features.

### 3.5 Hybridization approach

There are several reasons why hybridization is needed in deep learning models (Karasulu, Yücalar, & Borandag, 2022). Data limitations: In some cases, it can be difficult to have enough labeled data. This is especially true for datasets that require rare or expensive labeling processes. Hybridization can combine a small amount of labeled data with a larger unlabeled dataset, resulting in greater diversity and insight. This can help the model generalize better and produce better results. Scalability: Deep learning models generally perform better when trained on large datasets. However, labeled data collection and labeling processes are often time‐consuming and costly. By taking advantage of a larger unlabeled dataset, hybridization can increase the scalability of the model with more data and provide more general learning. Transfer learning: The transfer of previously learned information in one task to another task is called transfer learning. Hybridization can accelerate learning or achieve better success for another task using knowledge learned in one task. A pre‐trained deep learning model can capture general features and representations and use this information in another task with hybridization methods (Karasulu, Yücalar, & Borandağ, 2022). Learning confusion: Hybridization can help the model develop a more general understanding by combining different types of data. For example, a deep learning model for an image classification task can gain a more comprehensive understanding by integrating text data also through hybridization. This allows the model to explore more complex relationships and produce better results (Samee et al., 2022; Taspinar et al., 2022).

Based on the reasons for hybridizing the models, in this study, it is aimed to provide learning complexity and to achieve a higher level of learning and to increase the success of the newly created models. There is no rule that hybrid models will always perform better than single models. In this study, many models were tested, hybridized with different methods and the most successful models were presented in the study.

### 3.6 Confusion matrix

A confusion matrix is used to evaluate a classification model or compare it with different models (Kaya & Saritas, 2019; Taspinar et al., 2021b). The amount of data in the confusion matrix changes depending on the number of classes in the dataset. Rows or columns represent actual and predicted values (Koklu & Taspinar, 2021). A confusion matrix has TP (true positive), TN (true negative), FP (false positive), and FN (false negative) values for each class (Koklu et al., 2012; Kursun et al., 2022). In Table 2, the four‐class confusion matrix is shown. Table 3 gives the calculation of TP, TN, FP, and FN values.

**TABLE 2:** Four class confusion matrix.

Predicted	Actual			
	BT6470	CALIPOS	ES_ARMANDI	HIVA
BT6470	*T*_1_	*F*_12_	*F*_13_	*F*_14_
CALIPOS	*F*_21_	*T*_2_	*F*_23_	*F*_24_
ES_ARMANDI	*F*_31_	*F*_32_	*T*_3_	*F*_34_
HIVA	*F*_41_	*F*_42_	*F*_43_	*T*_4_

**TABLE 3:** Calculation of TP, TN, FP, and FN values according to classes.

BT6470	CALIPOS	ES_ARMANDI	HIVA
*TP*_1_ *= T*_1_ *TN*_1_ *= T*_2_ *+ T*_3_ *+ T*_4_ *+ F*_23_ *+ F*_24_ *+ F*_32_ *+ F*_34_ *+ F*_42_ *+ F*_44_ *FN*_1_ *= F*_21_ *+ F*_31_ *+ F*_41_ *FP*_1_ *= F*_12_ *+ F*_13_ *+ F*_14_	*TP*_2_ *= T*_2_ *TN*_2_ *= T*_1_ *+ T*_3_ *+ T*_4_ *+ F*_34_ *+ F*_43_ *+ F*_13_ *+ F*_14_ *+ F*_31_ *+ F*_41_ *FN*_2_ *= F*_12_ *+ F*_32_ *+ F*_42_ *FP*_2_ *= F*_21_ *+ F*_23_ *+ F*_24_	*TP*_3_ *= T*_3_ *TN*_3_ *= T*_1_ *+ T*_2_ *+ T*_4_ *+ F*_12_ *+ F*_21_ *+ F*_14_ *+ F*_24_ *+ F*_41_ *+ F*_42_ *FN*_3_ *= F*_13_ *+ F*_23_ *+ F*_43_ *FP*_3_ *= F*_31_ *+ F*_32_ *+ F*_34_	*TP*_4_ *= T*_4_ *TN*_4_ *= T*_1_ *+ T*_2_ *+ T*_3_ *+ F*_12_ *+ F*_13_ *+ F*_23_ *+ F*_21_ *+ F*_31_ *+ F*_32_ *FN*_4_ *= F*_14_ *+ F*_24_ *+ F*_34_ *FP*_4_ *= F*_41_ *+ F*_42_ *+ F*_43_

### 3.7 Performance evaluation

The performance of a classification model cannot be measured with a single metric (Borandağ et al., n.d.). Therefore, evaluations should be performed with more than one metric (Altay & Altay, 2023b). On the other hand, some metrics can be misleading in datasets with an unbalanced number of data (Cinar et al., 2022; Taspinar, 2022). Accuracy (ACC), recall (RCL), specificity (SPC), precision (PSC), and F1 score (FSC) are performance metrics frequently used in the literature (Singh et al., 2022; Taspinar, Cinar, & Koklu, 2021). The formulas of these metrics are given in Table 4.

**TABLE 4:** Performance metrics formulas.

Metrics	Equation
Accuracy	$\frac{\mathrm{TP}+\mathrm{TN}}{\mathrm{TP}+\mathrm{TN}+\mathrm{FP}+\mathrm{FN}}\times 100$
Precision	$\frac{\mathrm{TP}}{\mathrm{TP}+\mathrm{FP}}$
Recall	$\frac{\mathrm{TP}}{\mathrm{TP}+\mathrm{FN}}$
Specificity	$\frac{\mathrm{TN}}{\mathrm{TN}+\mathrm{FP}}$
F1 score	$2x\frac{\text{Precision}*\text{Recall}}{\text{Precision}+\text{Recall}}$

Performance metrics are used to evaluate the performance of classification models. However, it is important that each metric is evaluated according to its limitations and context. It can be more informative to use multiple metrics rather than a single metric, especially when unstable data sets or certain types of errors are important.

#### 3.7.1 Accuracy

Accuracy represents the percentage of samples that a classification model predicts correctly. It is calculated as the ratio of correctly predicted samples to total samples. Accuracy is used to evaluate overall model performance.

Limitations:

- If the dataset is unbalanced across classes (one class contains many more samples than the other), the accuracy metric can be misleading. The model is able to achieve high accuracy by focusing on the majority class.
- Accuracy does not provide information about false positives and false negatives. Therefore, it should be used with other metrics to understand the error types or to minimize a particular error type (Yasar, 2023b).

#### 3.7.2 Precision

Precision represents the percentage of samples predicted as positive and samples that are actually positive. It aims to reduce the number of false positives.

Limitations:

- Precision does not take into account false negatives (miss true positives). Therefore, precision alone is not an adequate metric when false negatives are significant.
- In the case of unstable data sets, the precision value can be misleading. To obtain high precision, the model may choose not to predict the rare class (Golcuk & Yasar, 2023).

#### 3.7.3 Recall or sensitivity

Recall represents the percentage of samples that are predicted as true positive, of samples that are actually positive. It aims to reduce false negatives (missing true positives).

Limitations:

- Recall ignores false positives. Where false positives are significant, recall alone is not an adequate metric.
- In unstable datasets, the rare class may be difficult to accurately predict and the recall may be low (Yasar, 2023c).

#### 3.7.4 F1 score (F1 score)

F1 score represents the harmonic mean of precision and recall metrics. The F1 score is used where the classification model aims to reduce both false positives and false negatives.

Limitations:

- In unstable datasets, the rare class may be difficult to accurately predict and the F1 score may be low.
- The F1 score is a combination of precision and recall metrics. However, in some cases, recall may decrease or increase while accuracy increases. Therefore, the best metric should be chosen for a particular scenario (Yasar, 2023c).

#### 3.7.5 Specificity

Represents the percentage of samples that are predicted as negative grades and samples that are actually negative. The specificity metric is an important metric, especially when the negative class needs to be protected from false positives.

Limitations:

- May be misleading when used on datasets with class imbalance. If the negative class is more common in the data set and the model focuses on the majority class, the specificity may be high, but the actual performance of the model may be low.
- Specificity provides information on accurately estimating the negative class, but does not evaluate the performance of the positive class. Therefore, using it alone is not enough. It should be used in conjunction with other metrics such as precision, recall, and F1 score (Gören & Çınarer, 2023).

## 4 EXPERIMENTAL RESULTS

In this chapter, the success of AlexNet and ResNet50 models for classification of the maize seeds and the models obtained by hybridization of these models with LSTM and BiLSTM are examined. The depth of the AlexNet model is 8, while the depth of the ResNet50 model is 50. Depth refers to the number of layers in CNN models. The input layer of the AlexNet model uses 227 × 227 × 3 pixel images, while the input layer of the ResNet50 model uses 224 × 224 × 3 pixel images as input. The experiments carried out within the scope of this study were performed on a computer with Intel® Core i7™ 12,700 K 3.61 GHz, NVIDIA GeForce RTX 3080Ti, and 64GB RAM. 80% of 14,469 images were used for training and 20% were used for testing. The parameters used for each model, the fine tunings made on the models, and the obtained results are given in separate titles. The parameters values used for the models in the classification are given in Table 5.

**TABLE 5:** Parameters used for transfer learning CNN models.

Model parameters	
Solver	SDGM
InitialLearnRate	0.001
Validation frequency	5
Max epochs	8
Execution environment	11

The choice of training parameters requires a balance. It is important to try different combinations of parameters to speed up the training process, prevent overfitting, manage memory requirements, and improve generalization performance. Practical experiments and dataset‐specific analyses play an important role in determining the best parameter setting. Factors such as data set properties, model complexity, and computational resources should be considered when choosing a solver. SGDM was chosen as the solver in this study because it is a simple algorithm and parameter update can be performed faster. The learning rate determines how many “steps” are taken at each step when updating the parameters of the network. Choosing the right learning rate affects the speed and stability of the educational process. Setting the learning rate as a large value may speed up the training process, but may exceed the point of convergence. Setting it to a small value may result in a slower training process, but will provide a more stable convergence. Considering these situations, the learning rate was determined as 0.0001. The epoch number determines how many times the network will train the dataset. A high epoch value may increase the risk of overfitting the model to the training data, as well as increase the training time and computational cost. Therefore, considering the size of the data set, max. The epoch value was set to 8. The mini‐batch size specifies the number of samples to be used in each step. Since mini‐batch size selection is effective on the speed of the training process, memory usage, and generalization performance, this value was chosen as 16 considering the device resources used. Momentum determines how much the gradient interacts with the previous step in the optimization algorithm. While high momentum can increase the risk of overfitting, it can provide rapid convergence. Low momentum can reduce the risk of overfitting and provide better generalization performance. Different values were tried for the momentum value suitable for the data set and the model, and the most appropriate value was determined as 0.9.

### 4.1 AlexNet results

**FIGURE 6:**
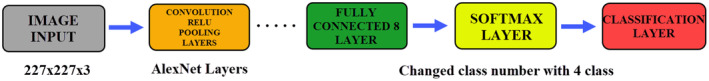
AlexNet architecture and fine‐tuning processes.

The parameters used for training the AlexNet model are as follows: Solver: SGDM (stochastic gradient descent with momentum), initial learn rate: 0.0001, validation frequency: 5, maximum epochs: 8, mini batch size: 16, execution environment: GPU, momentum: 0.9, learn rate drop factor. The data used in the study were classified by fine‐tuning the pre‐trained AlexNet model with the TL method. In the last fully connected layer of the AlexNet model, training and testing processes were carried out by setting the number of classes to 4. The general structure of the model and the changes made are shown in Figure 6.

**FIGURE 7:**
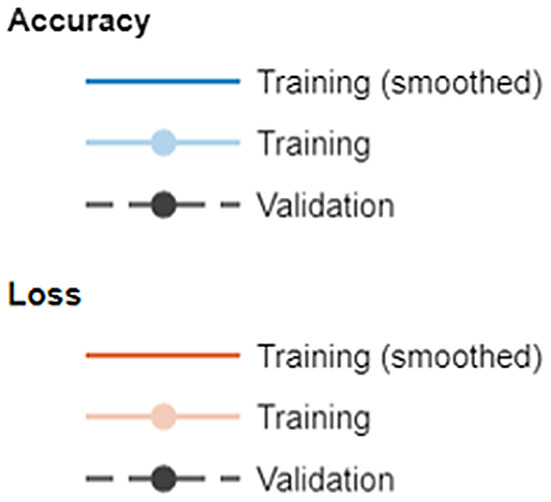
Descriptions of curves in charts.

The graphs obtained while training and testing the models contain information about the learning levels and estimation levels of the models. What the curves in the graphs mean is shown in Figure 7.

**FIGURE 8:**
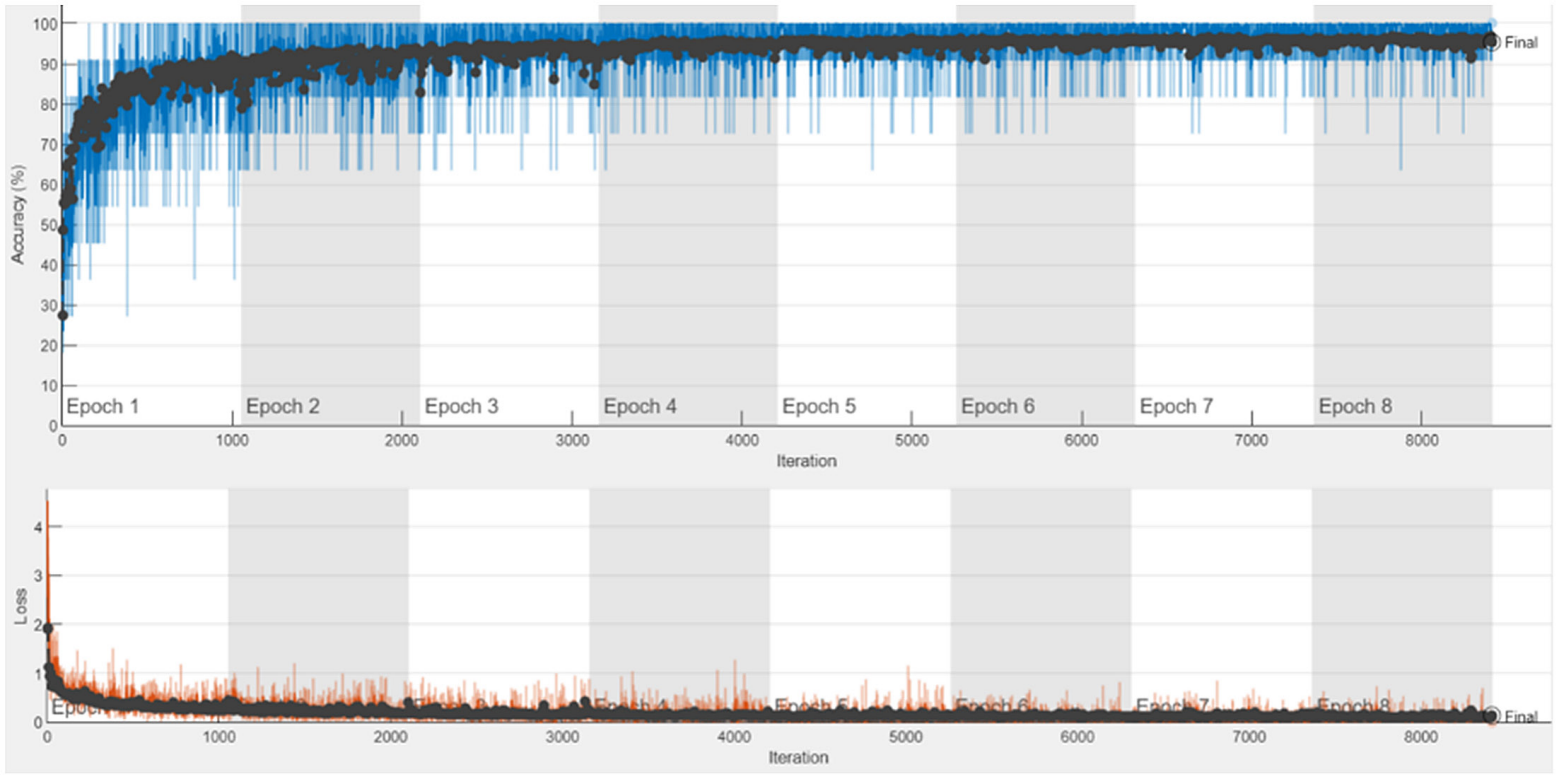
AlexNet accuracy and loss graph.

The training of the AlexNet model was carried out by using the transfer learning method. Figure 8 gives the accuracy and loss graphs obtained as a result of training and testing processes. The confusion matrix obtained as a result of the model's testing is shown in Table 6.

**TABLE 6:** Confusion matrix of AlexNet model.

	Actual class			
	BT6470	CALIPOS	ES_ARMANDI	HIVA
Predicted class				
BT6470	521	4	15	9
CALIPOS	2	1008	8	2
ES_ARMANDI	14	5	648	4
HIVA	74	1	6	573

Dark colors represent TP values and light colors represent the rest.

Using the confusion matrix data in Table 6, the AlexNet model's accuracy, precision, recall, and F1 score performance metrics were calculated according to classes. Table 7 gives the performance metrics of the AlexNet model.

**TABLE 7:** Performance metrics of AlexNet model.

	Accuracy	Precision	Recall	F1 score
BT6470	96.92	0.95	0.85	0.9
CALIPOS	99.24	0.99	0.99	0.99
ES_ARMANDI	98.2	0.97	0.96	0.96
HIVA	96.68	0.88	0.97	0.92

According to Table 5, higher success was achieved in the classification of the CALIPOS compared to other cultivars. In classification with the AlexNet model, it was seen that the CALIPOS is the least confused cultivar with the other cultivars. Precision, recall, and F1 score values are also the highest in CALIPOS class. The average classification success of the AlexNet model was calculated as 95.02%.

### 4.2 AlexNet+LSTM results

**FIGURE 9:**
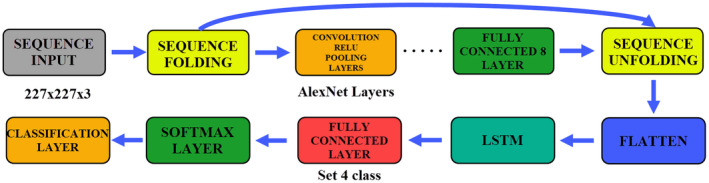
Structure and fine‐tuning processes of AlexNet+LSTM model.

There are many reasons for using LSTM as a hybrid with CNN models. One of these reasons is to enable the model to learn more by giving the data to the network as a sequence. Increasing the learning level of the network recursively with LSTM is also among these reasons. It is not expected that the network will increase the learning level in each data. In this study, LSTM was used as a hybrid with CNN models and the created models increased the classification success. While adding LSTM to CNN models, layer changes are made at the inputs and outputs of the models. The parameters of the AlexNet model are used in the same way. The LSTM parameters are as follows: Input size: Auto, Number of Hidden Units: 100, Output Mode: Last, State Activation Function: tanh, Gate Activation Function: sigmoid. In Figure 9, the creation of the AlexNet+LSTM model and the fine‐tuning processes are shown.

**FIGURE 10:**
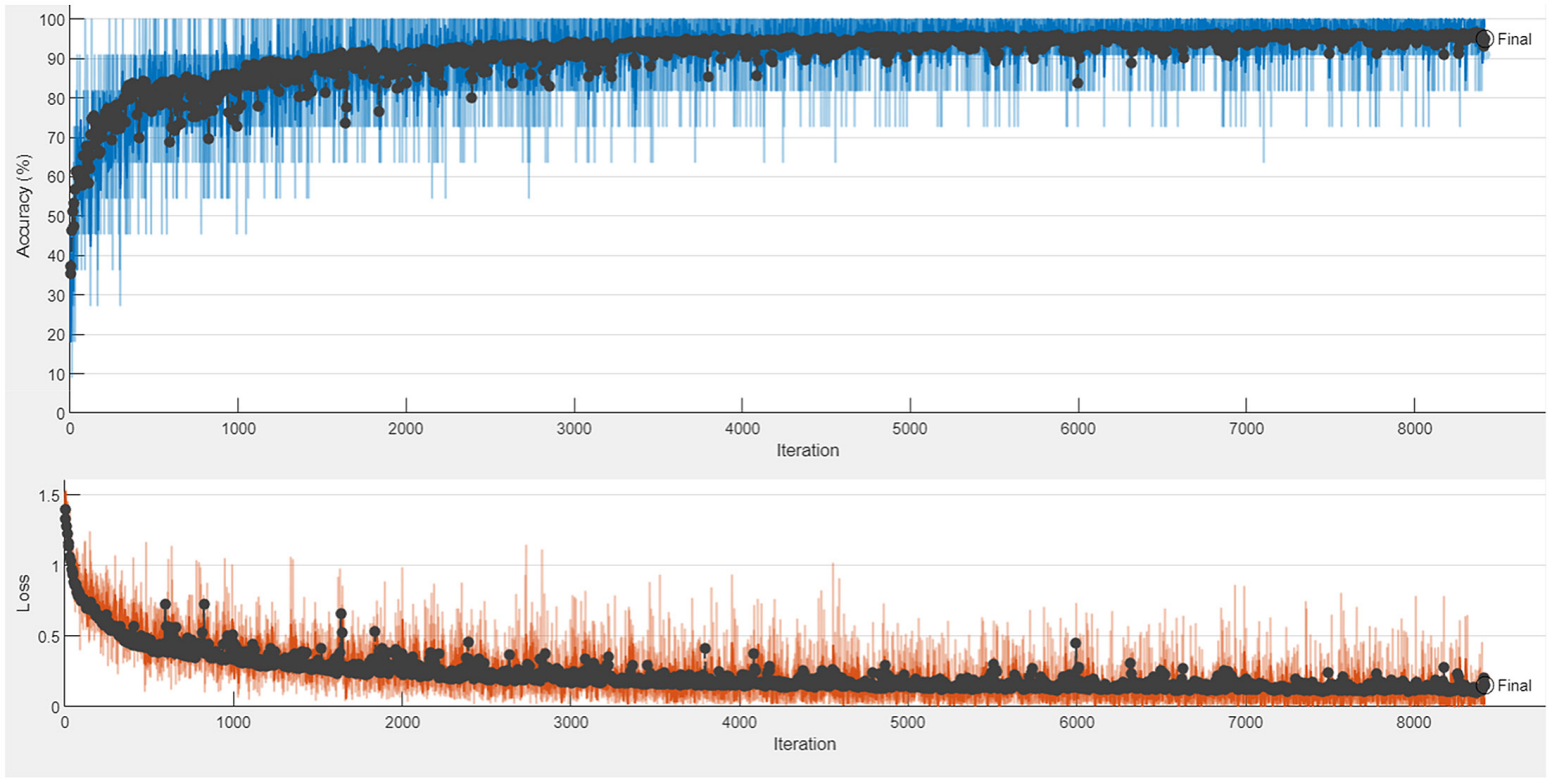
AlexNet+LSTM accuracy and loss graph.

Figure 10 gives the accuracy and loss graphs obtained as a result of training and testing the AlexNet+LSTM model. The confusion matrix obtained as a result of the test is shown in Table 8.

**TABLE 8:** Confusion matrix of AlexNet+LSTM model.

	Actual class			
	BT6470	CALIPOS	ES_ARMANDI	HIVA
Predicted class				
BT6470	524	0	16	11
CALIPOS	0	1014	6	0
ES_ARMANDI	11	4	648	1
HIVA	76	0	7	576

Dark colors represent TP values and light colors represent the rest.

Using the confusion matrix data in Table 8, accuracy, precision, recall, and F1 score performance metrics of the AlexNet+LSTM model were calculated according to classes. In addition, the classification numbers of the classes were added. The performance metrics of the AlexNet+LSTM model are given in Table 9.

**TABLE 9:** Performance metrics of AlexNet+LSTM model.

	Accuracy	Precision	Recall	F1 score
BT6470	96.06	0.95	0.86	0.9
CALIPOS	99.65	0.99	1	1
ES_ARMANDI	98.45	0.98	0.96	0.97
HIVA	96.72	0.87	0.98	0.92

According to Table 9, the CALIPOS cultivar was classified more successfully compared to other cultivars. It is seen that the CALIPOS is the least confused cultivar with the other cultivars. Precision, recall, and F1 score values are also the highest in CALIPOS class. The average classification success of the AlexNet model was calculated as 95.44%. As a result of the hybridization of the AlexNet model with LSTM, an increase was observed in the classification success of CALIPOS, ES_ARMANDI, and HIVA classes. LSTM was effective in increasing the average classification success.

### 4.3 AlexNet+BiLSTM results

**FIGURE 11:**
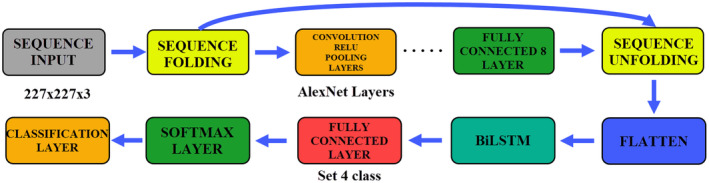
Architecture and fine‐tuning processes of AlexNet+BiLSTM.

The structure of AlexNet+BiLSTM is the same as the AlexNet+LSTM structure. The same parameters were used. In this model, the only layer that was changed is BiLSTM. BiLSTM contains a double LSTM structure. It increases classification success in many applications. It was used in this study to increase classification success. Figure 11 shows the stages of creating the AlexNet+BiLSTM model.

**FIGURE 12:**
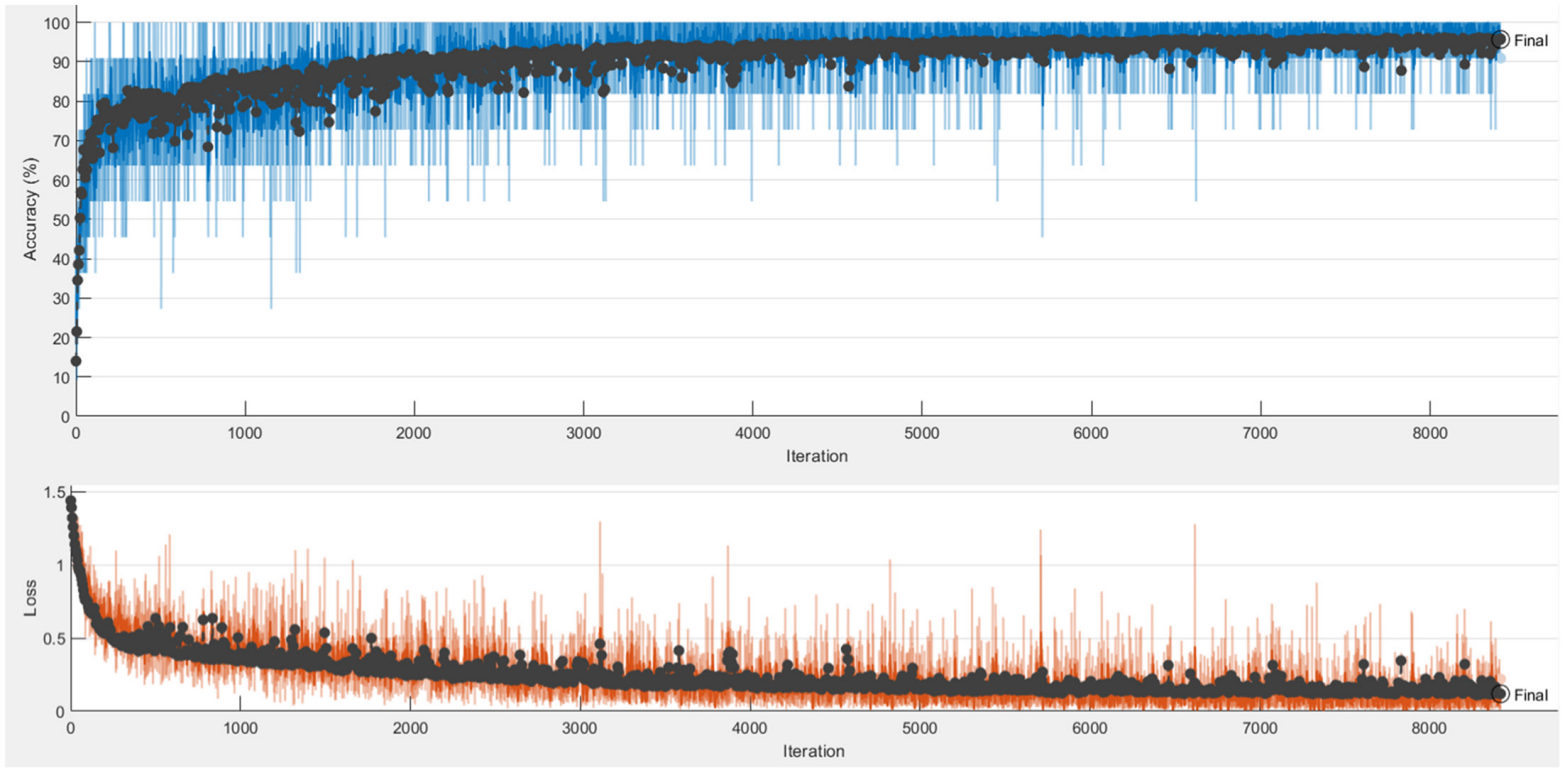
AlexNet+BiLSTM accuracy and loss graph.

Figure 12 demonstrates the accuracy and loss graphs obtained as a result of training and testing the AlexNet+BiLSTM model. The confusion matrix obtained as a result of the test is shown in Table 10.

**TABLE 10:** Confusion matrix of AlexNet+BiLSTM.

	Actual class			
	BT6470	CALIPOS	ES_ARMANDI	HIVA
Predicted class				
BT6470	528	2	12	6
CALIPOS	2	1011	8	2
ES_ARMANDI	12	3	652	3
HIVA	69	2	5	577

Dark colors represent TP values and light colors represent the rest.

Using the confusion matrix data in Table 10, the accuracy, precision, recall, and F1 score performance metrics of the AlexNet+BiLSTM model were calculated according to classes. In addition, the classification numbers of the classes were also added. The performance metrics of the AlexNet+BiLSTM model are given in Table 11.

**TABLE 11:** Performance metrics of AlexNet+BiLSTM.

	Actual	Predicted	Accuracy	Precision	Recall	F1 score
BT6470	611	548	96.44	0.96	0.86	0.91
CALIPOS	1018	1023	99.34	0.99	0.99	0.99
ES_ARMANDI	677	670	98.51	0.97	0.96	0.97
HIVA	588	653	96.99	0.88	0.98	0.93

According to Table 9, the CALIPOS cultivar was classified more successfully than other cultivars. Precision, recall, and F1 score values are also the highest in CALIPOS class. The average classification success of the AlexNet model was calculated as 95.65%. As a result of the hybridization of the AlexNet model with LSTM, an increase was observed in the classification success of BT6470, ES_ARMANDI, and HIVA classes. BiLSTM became effective in increasing the average classification success.

### 4.4 ResNet50 results

**FIGURE 13:**
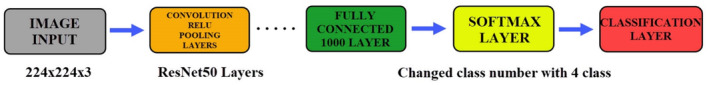
ResNet50 architecture and fine‐tuning processes.

The parameters used for training the ResNet50 model are as follows: Solver: SGDM (stochastic gradient descent with momentum), initial learn rate: 0.0001, validation frequency: 5, maximum epochs: 8, mini batch size: 16, execution environment: GPU, momentum: 0.9, learn rate drop factor: 0.1. By fine‐tuning the pre‐trained AlexNet model with the TL method, the data used in the study was classified. In the last fully connected layer of the ResNet50 model, training and testing processes were carried out by setting the number of classes to 4. The general structure of the model and the changes made are shown in Figure 13.

**FIGURE 14:**
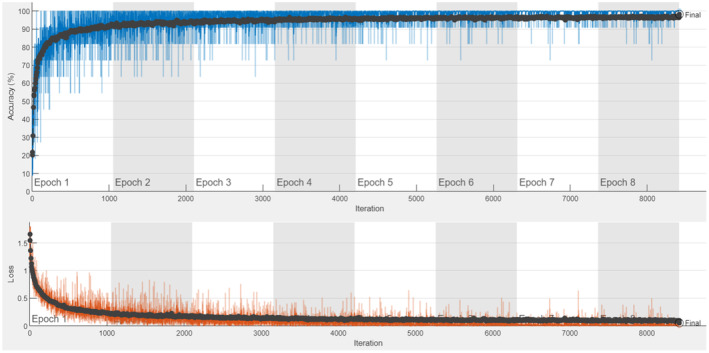
ResNet50 accuracy and loss graph.

The accuracy and loss graphs obtained as a result of training and testing the ResNet50 model are shown in Figure 14. The confusion matrix obtained as a result of the test is shown in Table 12.

**TABLE 12:** Confusion matrix of ResNet50 model.

	Actual class			
	BT6470	CALIPOS	ES_ARMANDI	HIVA
Predicted class				
BT6470	572	4	5	12
CALIPOS	4	1008	4	2
ES_ARMANDI	13	3	665	4
HIVA	22	3	3	570

Dark colors represent TP values and light colors represent the rest.

Accuracy, precision, recall, and F1 score performance metrics of the ResNet50 model according to classes were calculated by using the confusion matrix data in Table 12. In addition, the classification numbers of the classes were also added. The performance metrics of the ResNet50 model are given in Table 13.

**TABLE 13:** Performance metrics of ResNet50.

	Accuracy	Precision	Recall	F1 score
BT6470	97.93	0.96	0.94	0.95
CALIPOS	99.31	0.99	0.99	0.99
ES_ARMANDI	98.89	0.97	0.98	0.98
HIVA	98.41	0.95	0.97	0.96

According to Table 11, the CALIPOS cultivar was classified more successfully than other cultivars. BT6470 is the class with the lowest classification success. Precision, recall, and F1 score values are also the highest in CALIPOS class.

### 4.5 ResNet50+LSTM results

**FIGURE 15:**
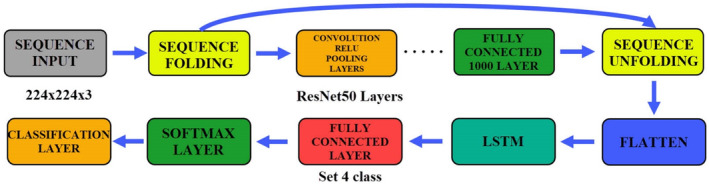
ResNet50+LSTM architecture and fine‐tuning processes.

The parameters of the ResNet50 model used in this section were kept the same. The LSTM parameters are as follows: Input size: auto, number of hidden units: 100, output mode: last, state activation function: tanh, gate activation function: sigmoid. The creation of the ResNet50+LSTM model and the fine‐tuning processes are shown in Figure 15.

**FIGURE 16:**
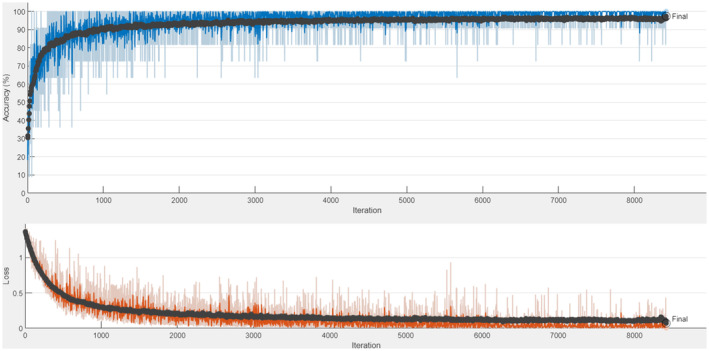
ResNet50+LSTM accuracy and loss graph.

The accuracy and loss graphs obtained as a result of training and testing the ResNet50+LSTM model are shown in Figure 16. The confusion matrix obtained as a result of the test is shown in Table 14.

**TABLE 14:** Confusion matrix of ResNet50 + LSTM model.

	Actual class			
	BT6470	CALIPOS	ES_ARMANDI	HIVA
Predicted class				
BT6470	575	3	12	6
CALIPOS	2	1008	4	1
ES_ARMANDI	12	4	666	4
HIVA	22	3	2	572

Dark colors represent TP values and light colors represent the rest.

Accuracy, precision, recall, and F1 score performance metrics of the ResNet50+LSTM model were calculated according to classes by using the confusion matrix data in Table 14. In addition, the classification numbers of the classes were also added. The performance metrics of the ResNet50+LSTM model are given in Table 15.

**TABLE 15:** Performance metrics of ResNet50 + LSTM.

	Accuracy	Precision	Recall	F1 score
BT6470	98.1	0.97	0.94	0.95
CALIPOS	99.41	0.99	0.99	0.99
ES_ARMANDI	98.93	0.97	0.98	0.98
HIVA	98.51	0.95	0.97	0.96

According to Table 13, the CALIPOS cultivar was classified more successfully than other cultivars. Precision, recall, and F1 score values are also highest in the CALIPOS class. The average classification success of the ResNet50+LSTM model was calculated as 97.48%. As a result of the hybridization of the ResNet model with LSTM, an increase was observed in the classification success of BT6470, CALIPOS, ES_ARMANDI, and HIVA classes compared to the ResNet50 model. LSTM became effective in increasing the average classification success.

### 4.6 ResNet50+BiLSTM results

**FIGURE 17:**
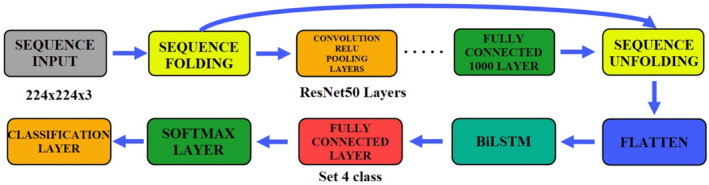
ResNet50+BiLSTM architecture and fine‐tuning processes.

The structure of ResNet50+BiLSTM is the same as the AlexNet+LSTM structure. The same parameters were used. In this model, only the BiLSTM layer was changed. BiLSTM contains a double LSTM structure. It increases classification success in many applications. It was used in this study to increase classification success. Figure 17 shows the stages of creating the ResNet50+BiLSTM model.

**FIGURE 18:**
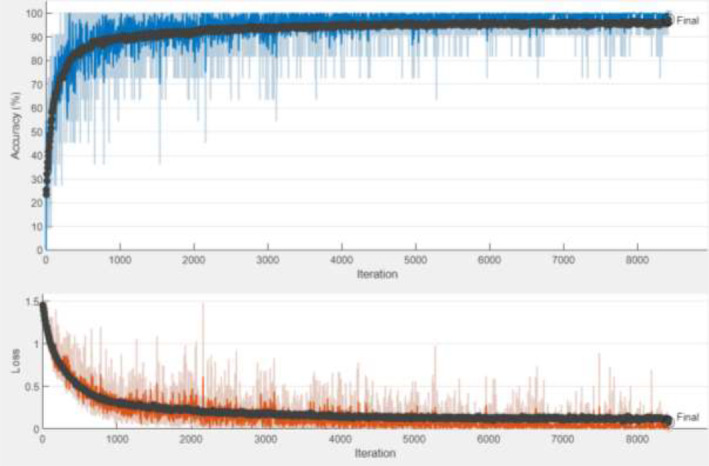
ResNet50+BiLSTM accuracy and loss graph.

Figure 18 shows the accuracy and loss graphs obtained as a result of training and testing the ResNet50+BiLSTM. The confusion matrix obtained as a result of the test is shown in Table 16.

**TABLE 16:** Confusion matrix of ResNet50 + BiLSTM model.

	Actual class			
	BT6470	CALIPOS	ES_ARMANDI	HIVA
Predicted class				
BT6470	581	0	3	14
CALIPOS	0	1018	4	0
ES_ARMANDI	11	0	670	4
HIVA	19	0	0	570

Dark colors represent TP values and light colors represent the rest.

Accuracy, precision, recall, and F1 score performance metrics of the ResNet50+LSTM model were calculated according to classes by using the confusion matrix data in Table 16. In addition, the classification numbers of the classes were also added. The performance metrics of the ResNet50+BiLSTM model are given in Table 17.

**TABLE 17:** Performance metrics of ResNet50 + BiLSTM.

	Accuracy	Precision	Recall	F1 score
BT6470	98.38	0.97	0.95	0.96
CALIPOS	99.86	1.00	1.00	1.00
ES_ARMANDI	99.24	0.98	0.99	0.98
HIVA	98.72	0.97	0.97	0.97

According to Table 15, the CALIPOS cultivar was classified more successfully than other cultivars. Precision, recall, and F1 score values are also the highest in CALIPOS class. The average classification success of the ResNet50+BiLSTM model was calculated as 98.10%. As a result of the hybridization of the ResNet model with BiLSTM, an increase was observed in the classification success of BT6470, CALIPOS, ES_ARMANDI, and HIVA classes compared to the ResNet50+LSTM model. BiLSTM became effective in increasing the average classification success.

## 5 DISCUSSION

Although maize cultivars are normally similar to each other, they can be distinguished by their differences when viewed carefully by an expert person. But some maize cultivars may sometimes not be easily distinguished even by an expert person. In this study, automatic classification of maize kernels was carried out with CNN models, which are a sub‐branch of artificial intelligence developed inspired by human characteristics. Even deep learning models may not be able to give 100% results in classification processes, as in this study. As a result, the goals of researchers in classification problems are to try to bring this classification success closer to the highest level, 100% classification success.

The AlexNet and ResNet50 models selected from the pre‐trained models in the study are different models from each other in terms of depth and number of layers. For this reason, they show different classification success even in the same classification processes. The classification achievements of the selected models were developed as hybrid models and their performance in classification success was given in detail in the Experimental results section. The effect of LSTM and BiLSTM architectures, which are usually used in the processing of time‐dependent data in the hybridization of models, on image classification problems was investigated with this study. The LSTM architecture adds a memory layer to the model, and the BiLSTM architecture, in addition to adding a memory layer, allows both past and future connections to be taken into account differently. It allows both hybridization models to be used more effectively.

In this study, maize seeds were classified with six different CNN models. Classification successes were achieved as a result of the training and testing processes of the models, AlexNet, AlexNet+LSTM, AlexNet+BiLSTM, ResNet50, ResNet50+LSTM, and ResNet+BiLSTM models. The classification success of all models is shown in Table 18.

**TABLE 18:** Comparison of classification accuracy for all models.

	AlexNet	AlexNet+ LSTM	AlexNet+ BiLSTM	ResNet50	ResNet50+ LSTM	ResNet50+ BiLSTM
Accuracy	95.02	95.44	95.65	97.27	97.48	98.10

According to Table 16, the AlexNet model has the lowest classification success. The average classification success achieved with the AlexNet+LSTM model obtained as a result of the hybridization of this model with LSTM is higher compared to the AlexNet model. The AlexNet+BiLSTM model obtained by hybridizing the AlexNet model with BiLSTM also achieved a higher average classification success than the AlexNet+LSTM model. In the light of these results, it can be stated that the hybridization of the AlexNet model increases the average classification success.

As a result of the classification performed with the ResNet50 model, a higher classification success was obtained compared to the AlexNet+BiLSTM model. It is thought that this is because the number of ResNet50 models' layers is higher than the AlexNet and AlexNet hybrid models. The high number of layers does not always increase classification success. However, in this study, higher classification success was obtained from the ResNet50 model and its hybrids, which have more layers in comparison with the AlexNet and its hybrids. The ResNet50+LSTM model, obtained as a result of hybridizing the ResNet50 model with LSTM, is more successful than the ResNet50 model in terms of average classification success. The ResNet50+BiLSTM model, which was created as a result of the hybridization of the ResNet50 model with BiLSTM, was also more successful in terms of average classification success compared to the ResNet50+LSTM model. In this case, it can be stated that the hybridization of the models in this study increases the average classification success. Therefore, in line with the results of this study, it can be stated that the hybridization of the models increases the average classification success. In addition, it is seen that the number of layers has an impact on the average classification success.

## 6 CONCLUSIONS

For this study, a dataset containing a total of 14,469 maize seed images in four classes was created. Six different CNN‐based models were proposed for the classification of maize seeds in the created dataset. AlexNet and ResNet50 pre‐trained models were used for both classification and the creation of hybrid models. LSTM and BiLSTM were utilized to hybridize CNN models. As a result, AlexNet, AlexNet+LSTM, AlexNet+BiLSTM, ResNet50, ResNet50+LSTM, and ResNet50+BiLSTM models were created to classify the images of maize seeds. The ranking of the models' classification successes is also in the same way. The lowest classification success belongs to the AlexNet model, while the highest classification success belongs to the ResNet50+LSTM model. The classification success of the ResNet50+BiLSTM model is 98.10%.

Pre‐trained models ensure fast results in classification studies. In this study, in addition to the AlexNet and ResNet50 models from the pre‐trained models, the LSTM and BiLSTM architectures were added to the models and hybridized. Although both hybrid models have given more successful results than the plain model, they have brought cost and model confusion. However, the study also emphasizes that in cases where decation is important, cost and complexity can be ignored. Considering the pricing effect of a pure agricultural product, the maximum cost for classification success was tried in this study, and a positive result was obtained.

The classification success of the proposed models is sufficient for classifying maize seeds. Proposed models can be converted into lite (low‐dimensional) models and converted into applications that can be used in mobile operating systems. With these proposed models, maize seed cultivars can be determined in a quick and non‐destructive way. In the future, sorting different types of maize will be possible by integrating the models into delta robot systems. It is predicted that the separation of maize seed cultivars may be effective in improving efficiency by increasing the purity of the seeds. Commercially, the purity of maize seeds provides convenience in pricing.

## AUTHOR CONTRIBUTIONS

**Hakan Isik:** Formal analysis (equal); funding acquisition (equal). **Sakir Tasdemir:** Investigation (equal); methodology (equal); validation (equal); visualization (equal). **Yavuz Selim Taspinar:** Conceptualization (equal); data curation (equal); visualization (equal). **Ramazan Kursun:** Data curation (equal); formal analysis (equal); funding acquisition (equal). **Ilkay Cinar:** Methodology (equal); project administration (equal); visualization (equal). **Ali Yasar:** Software (equal); supervision (equal). **Elham Tahsin Yasin:** Investigation (equal); writing – original draft (equal). **Murat Koklu:** Validation (equal); visualization (equal).

## FUNDING INFORMATION

This study was supported by the Turkish Scientific and Technical Research Council (TUBITAK).

## CONFLICT OF INTEREST STATEMENT

The authors declare that they have no conflict of interest.

## ETHICS STATEMENT

The article does not contain any studies with human or animal subjects.

## Data Availability

The data can be downloaded from the link: https://muratkoklu.com/datasets/Maize_Image_Dataset.zip.
